# Thrombopoietin Contributes to Enhanced Platelet Activation in Patients with Type 1 Diabetes Mellitus

**DOI:** 10.3390/ijms22137032

**Published:** 2021-06-29

**Authors:** Ornella Bosco, Barbara Vizio, Gabriella Gruden, Martina Schiavello, Bartolomeo Lorenzati, Paolo Cavallo-Perin, Isabella Russo, Giuseppe Montrucchio, Enrico Lupia

**Affiliations:** 1 Department of Medical Sciences, University of Turin, 10126 Turin, Italy; ornella.bosco@unito.it (O.B.); barbara.vizio@unito.it (B.V.); gabriella.gruden@unito.it (G.G.); martina.schiavello@unito.it (M.S.); paolo.cavalloperin@gmail.com (P.C.-P.); 2 Emergency Medicine, A.O. S. Croce e Carle, 12100 Cuneo, Italy; lorebato@gmail.com; 3 Department of Clinical and Biological Sciences, University of Turin, 10043 Orbassano, Italy

**Keywords:** type 1 diabetes mellitus, platelet–leukocyte adhesion, platelet activation markers, thrombopoietin, atherosclerotic cardiovascular diseases

## Abstract

Atherosclerotic cardiovascular disease is the major cause of morbidity and mortality in patients with type 1 diabetes mellitus (T1DM). Enhanced platelet reactivity is considered a main determinant of the increased atherothrombotic risk of diabetic patients. Thrombopoietin (THPO), a humoral growth factor able to stimulate megakaryocyte proliferation and differentiation, also modulates the response of mature platelets by enhancing both activation and binding to leukocytes in response to different agonists. Increased THPO levels have been reported in different clinical conditions characterized by a generalized pro-thrombotic state, from acute coronary syndromes to sepsis/septic shock, and associated with elevated indices of platelet activation. To investigate the potential contribution of elevated THPO levels in platelet activation in T1DM patients, we studied 28 T1DM patients and 28 healthy subjects. We measured plasma levels of THPO, as well as platelet-leukocyte binding, P-selectin, and THPO receptor (THPOR) platelet expression. The priming activity of plasma from diabetic patients or healthy subjects on platelet–leukocyte binding and the role of THPO on this effect was also studied in vitro. T1DM patients had higher circulating THPO levels and increased platelet–monocyte and platelet–granulocyte binding, as well as platelet P-selectin expression, compared to healthy subjects, whereas platelet expression of THPOR did not differ between the two groups. THPO concentrations correlated with platelet–leukocyte binding, as well as with fasting glucose and Hb1Ac. In vitro, plasma from diabetic patients, but not from healthy subjects, primed platelet–leukocyte binding and platelet P-selectin expression. Blocking THPO biological activity using a specific inhibitor prevented the priming effect induced by plasma from diabetic patients. In conclusion, augmented THPO may enhance platelet activation in patients with T1DM, potentially participating in increasing atherosclerotic risk.

## 1. Introduction

Cardiovascular disease (CVD) is the major cause of morbidity and mortality in patients with type 1 diabetes mellitus (T1DM) [1]. The rising global incidence of T1DM with an even earlier age of onset leads to a longer exposure to the disease and a greater risk of premature CVD with microvascular and macrovascular complications that accelerate the process of atherosclerosis [2,3].

Pro-thrombotic factors, together with other pathophysiologic mechanisms, which include hyperglycemia, insulin resistance, hyperlipidemia, and a generalized pro-inflammatory state, play a determinant role in sustaining the accelerated atherosclerosis and increased cardiovascular risk profile of diabetic patients [4,5]. Enhanced platelet reactivity, together with endothelial dysfunction, increased levels of coagulation factors, and impaired fibrinolysis, has been associated with the elevated atherothrombotic risk of diabetic patients, and oral antiplatelet therapy has become a key treatment for reducing the long-term risk of ischemic events in DM patients [6,7,8]. Interestingly, DM patients remain at high risk of major cardiovascular events even on antiplatelet treatment, probably because of a greater prevalence, compared to non-diabetics, of inadequate platelet inhibition or “resistance” to antiplatelet therapy. The use of more potent and prolonged antiplatelet therapies reduces the rates of drug resistance and the recurrence of ischemic events, but the increase in bleeding complications represents a major concern [9].

Thrombopoietin (THPO) is a humoral growth factor constitutively produced by the liver and kidneys and promotes megakaryocyte proliferation and differentiation. The binding with its receptor c-Mpl (THPOR), mainly expressed on platelets and megakaryocytes, clears THPO from circulation and allows it to exert its biological functions [10,11].

Previous studies demonstrated that THPO directly modulates the response of mature platelets to several stimuli and thereby their homeostatic potential [12,13]. In particular, THPO, does not induce platelet aggregation per se, but can enhance both platelet activation and platelet–leukocyte adhesion in response to various agonists [12,13,14]. Increased THPO levels have been shown in different clinical conditions characterized by a pro-thrombotic state, from acute coronary syndromes to sepsis/septic shock and cigarette smoking, and are associated with elevated indices of platelet activation [15,16,17].

The aim of this study was to explore the potential contribution of THPO in enhancing platelet reactivity in patients with T1DM.

## 2. Results

### 2.1. Study Subjects

We enrolled 28 type 1 diabetic patients and 28 healthy subjects, who were matched in a 1:1 ratio for age and gender to cases.

Table 1 shows demographic and clinical data of both patients and controls.

Type 1 diabetic patients and healthy subjects did not differ regarding demographic characteristics. Platelet, leukocyte, monocyte, and granulocyte counts were not significantly different in the two groups, neither were mean platelet volume (MPV) and platelet distribution width (PDW). Finally, total, HDL and LDL cholesterol levels and triglycerides were similar between the two groups, whereas type 1 diabetic patients had, as expected, higher fasting glucose and HbA1c concentrations than healthy subjects.

### 2.2. THPO Concentrations and Platelet THPOR Expression in Type 1 Diabetes Mellitus Patients

**Figure 1:**
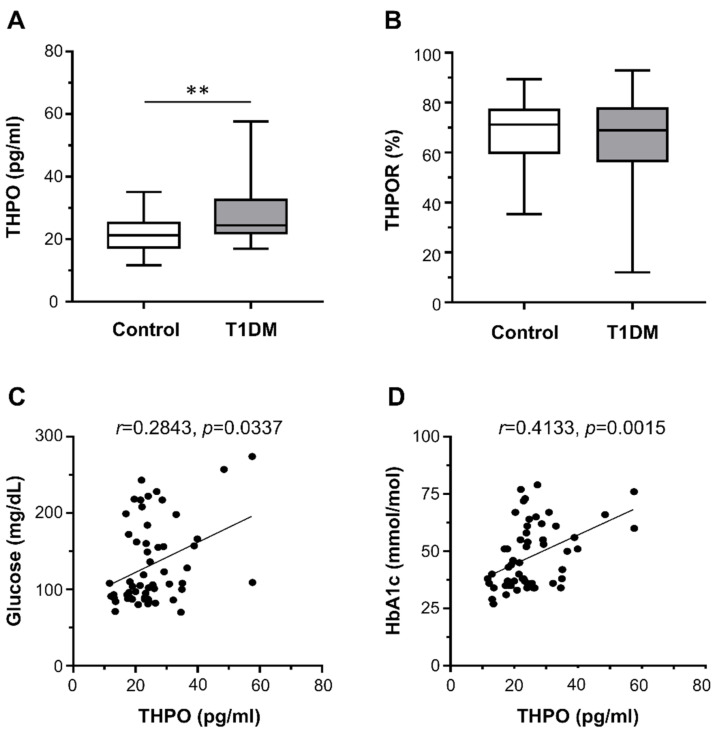
Circulating thrombopoietin (THPO) levels, measured by ELISA (**A**), and THPO receptor (R) expression, detected ex vivo by flow cytometry (**B**), in healthy controls (*n* = 28) and type 1 diabetes mellitus (T1DM) patients (*n* = 28). Data were represented as median (range). *p*-values by Mann–Whitney test ((**A**) ** *p* < 0.01; (**B**) not statistically significant). Correlation of THPO plasma levels with fasting plasma glucose (**C**) and HbA1c (**D**). Analyses by Spearman correlation tests; correlation coefficient r- and *p*-values are shown.

Plasma THPO concentrations were significantly higher in patients with T1DM than in healthy controls (median (range): 24.45 pg/mL (16.97–57.60) vs. 21.23 pg/mL (11.70–35.09)) (Figure 1A). In contrast, there were no differences in the surface expression of THPOR on platelets between T1DM patients and controls (median (range): 68.95% (12.00–92.90%) vs. 71.20% (35.40–89.40%)) (Figure 1B).

We found no correlation between THPO concentrations and either platelet count or markers of augmented platelet turnover (MPV and PDW). On the contrary, THPO concentrations positively correlated with both fasting plasma glucose (*r* = 0.2843, *p* = 0.0337; Figure 1C) and HbA1c (*r* = 0.4133, *p* = 0.0015; Figure 1D).

### 2.3. Ex Vivo Platelet Activation Studies

**Figure 2:**
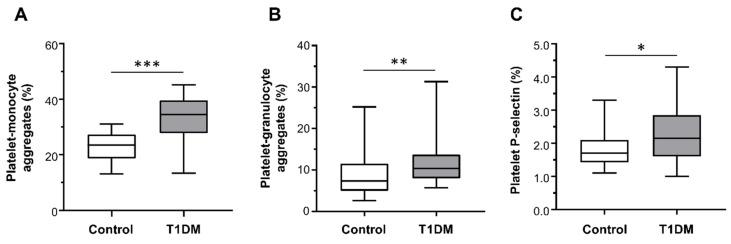
Platelet–monocyte aggregates (**A**), platelet–granulocyte aggregates (**B**), and platelet P-selectin expression (**C**) detected ex vivo, by flow cytometry, in healthy controls (*n* = 28) and type 1 diabetes mellitus (T1DM) patients (*n* = 28). Data were represented as median (range). *p*-values by Mann–Whitney test ((**A**) *** *p* < 0.001, (**B**) ** *p* < 0.01, and (**C**) * *p* < 0.05).

Patients with T1DM had significantly higher percentages of platelet–monocyte (median (range): 34.50% (13.40–45.20%) vs. 23.50% (13.10–31.00%); Figure 2A) and platelet–granulocyte binding (median (range): 10.35% (5.70–31.30%) vs. 7.35% (2.60–25.20%); Figure 2B), as well as increased surface expression of P-selectin on platelets (median (range): 2.15% (1.00–4.30%) vs. 1.70% (1.10–3.30%); Figure 2C) compared with healthy subjects.

**Figure 3:**
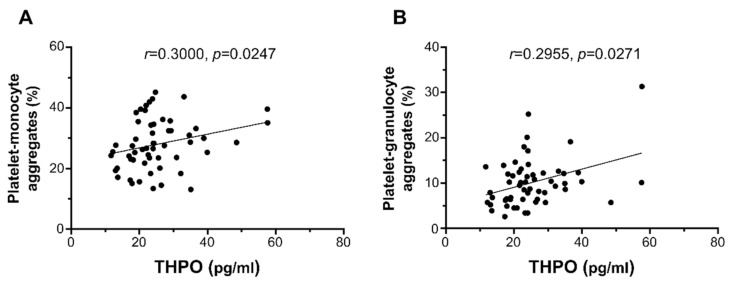
Correlation of THPO plasma levels with ex vivo platelet–monocyte (**A**) and platelet–granulocyte (**B**) aggregates. Analyses by Spearman correlation tests; correlation coefficient r- and *p*-values are shown.

THPO plasma levels significantly correlated with ex vivo platelet–monocyte aggregation (*r* = 0.3000, *p* = 0.0247; Figure 3A) and platelet–granulocyte binding (*r* = 0.2955, *p* = 0.0271; Figure 3B), but not with platelet P-selectin expression (*r* = 0.2411, *p* = 0.0735; not shown).

Moreover, fasting plasma glucose significantly correlated with ex vivo platelet–monocyte (*r* = 0.4104, *p* = 0.0017) and platelet–granulocyte aggregates (*r* = 0.2662, *p* = 0.0473), but not with platelet P-selectin expression. Finally, we observed a positive correlation between HbA1c and ex vivo platelet–monocyte aggregation (*r* = 0.5270, *p* < 0.0001), platelet–granulocyte binding (*r* = 0.3924, *p* = 0.0028), and platelet P-selectin expression (*r* = 0.3023, *p* = 0.0236).

### 2.4. Effect of Plasma of Type 1 Diabetes Mellitus Patients on Platelet Activation In Vitro

In order to explore the potential involvement of humoral mediators present in the plasma of diabetic patients in enhancing platelet activation, we tested in vitro the effect of plasma from diabetic patients and normal subjects on platelet–leukocyte adhesion in whole blood obtained from healthy donors.

Neither plasma from diabetic patients nor from healthy subjects increased platelet–leukocyte binding in whole blood per se, as determined by flow cytometric analysis.

**Figure 4:**
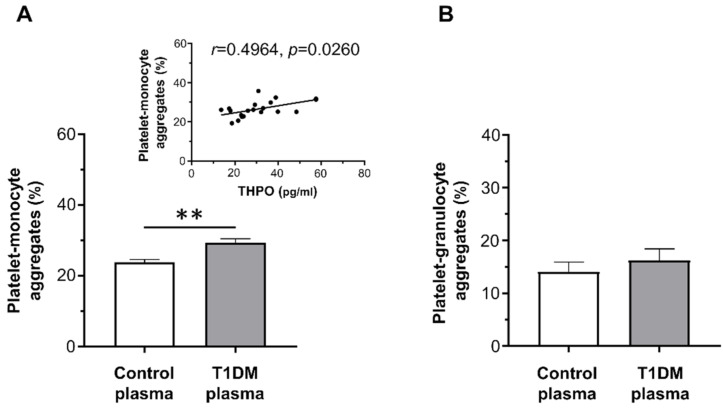
In vitro effect of plasma from healthy subjects and type 1 diabetic patients on epinephrine (EPI)-induced platelet–monocyte (**A**) and platelet–granulocyte binding (**B**), as analyzed by flow cytometry in whole blood. Data were represented as mean ± SE. *p*-values by unpaired *t*-test ((**A**) ** *p* < 0.01; (**B**) not statistically significant).

On the contrary, whereas plasma from healthy controls did not increase platelet–leukocyte aggregation, plasma from T1DM patients significantly enhanced platelet–monocyte binding induced by EPI (mean ± SE: 29.32% ± 1.11% vs. 23.80% ± 0.79%; Figure 4A), but not platelet–granulocyte binding (mean ± SE: 16.22% ± 2.21% vs. 14.08% ± 1.84%; Figure 4B).

THPO levels measured in vivo significantly correlated with the priming activity exerted in vitro by plasma samples from diabetic patients on platelet–monocyte aggregation (*r* = 0.4964, *p* = 0.0260; Figure 4A-insert).

**Figure 5:**
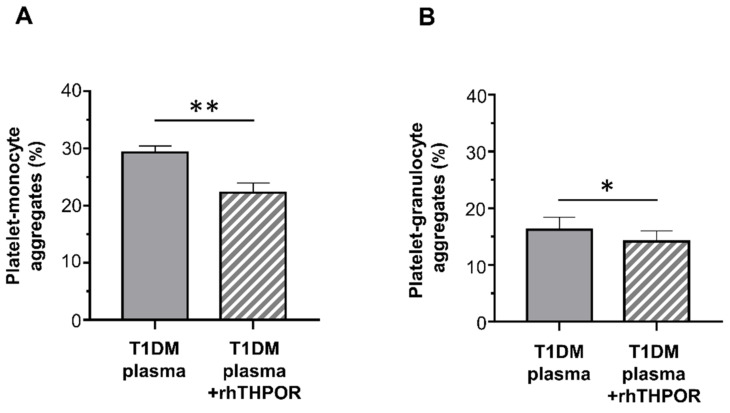
Effect of recombinant human (rh)THPOR on the priming activity induced by plasma from type 1 diabetic patients on EPI-induced platelet–monocyte (**A**) and platelet–granulocyte binding (**B**), as analyzed by flow cytometry in whole blood. Data were represented as mean ± SE. *p*-values by paired *t*-test ((**A**) ** *p* < 0.01; (**B**) * *p* < 0.05).

The contribution of THPO to this effect was assessed by inhibiting its biological activity using a recombinant human (rh)THPOR. Pre-treatment of plasma from T1DM patients with the rhTHPOR reduced the priming effect exerted by plasma from type 1 diabetic patients both on platelet–monocyte (mean ± SE: 22.24% ± 1.71% vs. 29.32% ± 1.11%; Figure 5A) and platelet–granulocyte binding (mean ± SE: 14.16% ± 1.85% vs. 16.22% ± 2.21%; Figure 5B). On the contrary, neither pre-incubation of plasma from healthy subjects with rhTHPOR nor rhTHPOR alone had a significant effect on platelet–leukocyte adhesion (data not shown).

## 3. Discussion

The results of our study suggest that THPO may participate in the pathophysiology of enhanced platelet activation in patients with type 1 diabetes mellitus. We found, indeed, that higher concentrations of circulating THPO were associated with increased indices of platelet activation in vivo in type 1 diabetic patients. Moreover, plasma of type 1 diabetic patients exerted in vitro a priming activity on platelet–leukocyte interaction that appeared to be mediated by plasma THPO contents.

In this study, we have shown that patients with type 1 diabetes mellitus had increased platelet–monocyte and platelet–granulocyte aggregates, together with augmented platelet P-selectin expression, compared to healthy subjects. Our present findings are in line with and confirm previous results demonstrating that type 1 diabetic patients have higher platelet reactivity, which may represent a potential mechanism leading to increased cardiovascular risk [18,19,20,21,22]. In fact, platelet–leukocyte aggregation, which is considered a sensitive measure of platelet activation, also has significant pro-inflammatory and pro-thrombotic consequences, in particular in acute coronary syndrome [23] and sepsis [16,24].

In our study, we also showed that type 1 diabetic patients had a significant, although not dramatic, increase in the circulating levels of THPO. The entity of THPO elevation was modest, as also confirmed by the absence of change in the expression of THPOR on platelet surface. However, since THPO is known to act in cooperation and/or synergy with other mediators, both on platelet activation and in other experimental contests [25], it is reasonable to hypothesize that even slight changes in THPO concentrations may be sufficient to influence these processes.

The description of increased THPO concentrations in type 1 diabetic patients is an innovative result, although the precise origin of the rise in THPO levels in type 1 diabetic patients cannot be definitely clarified.

We think that the observed increase in THPO levels in diabetic patients may probably depend on increased hepatic synthesis sustained by the chronic pro-inflammatory state present in diabetic patients and driven by other inflammatory humoral mediators, for instance, interleukin-6, the main acute-phase reactant produced in the liver [26], which is known to stimulate THPO synthesis in hepatocytes [27].

Much data indicate that THPO levels are primarily regulated by platelet mass [10,11]. However, the absence of thrombocytopenia in our patient population and the lack of correlation with MPV and PDW, which are considered markers of increased platelet turn-over, seem to exclude this causative mechanism in patients with type 1 diabetes mellitus. Still, previous studies, in populations larger than ours, have shown that diabetic patients have increased indices of platelet turn-over [28,29,30], and the consequent increased production of larger and more reactive new platelets has been related to increased platelet reactivity [31].

Finally, since we found a positive correlation between THPO and both platelet–leukocyte binding and platelet P-selectin expression in vivo, it can also be hypothesized that the augmented THPO concentrations are, at least partially, due to increased release by platelets themselves. Activated platelets, indeed, are known to release full-length biological active THPO upon stimulation [32]. Of note, THPO released by activated platelets may also concur to further increase inflammation by activating other cell types, for instance, stimulating motility and angiogenesis in endothelial cells [33] and enhancing interleukin-8 and reactive oxygen species release from neutrophils [34].

Several studies have shown that platelet activation is augmented in diabetic patients [35,36,37,38,39,40]. The pathophysiologic mechanisms involved in this phenomenon, although not fully elucidated, include, for instance, (a) the reduced production and activity of nitric oxide induced by oxidative stress [6,41,42]; (b) the generalized status of systemic hyper-inflammation, sustained by different mediators, present in diabetic patients [43,44]; and (c) qualitative and quantitative changes in coagulation factors [45,46], which contribute to the pro-thrombotic environment through the formation of compact fibrin networks and depressed fibrinolytic activity [47,48].

A main factor contributing to the enhanced platelet reactivity is represented by metabolic factors. Both acute and chronic changes in plasma glucose correlate, indeed, with increased expression of platelet activation markers, such as P-selectin and CD40-ligand, on platelet surface [6,49,50,51,52,53]. Hyperglycaemia may contribute to increase platelet activation via different mechanisms, which include augmented glycation of platelet surface proteins, which decreases membrane fluidity and increases platelet adhesion [54,55], and the direct osmotic effect of glucose [56], which can activate protein kinase C [57] and the nitric oxide/cyclic nucleotide pathway [58,59]. Further confirming these pieces of evidence, early intensive glucose control decreases platelet reactivity in patients with acute coronary syndrome and hyperglycaemia [60], and it reduces mortality in diabetic patients with acute coronary syndrome at 3.4 years of follow-up [61].

In our study, we found a significant correlation between THPO concentrations and both fasting glucose and HbA1c levels. These data confirm the causal relationship between metabolic control and platelet activation in type 1 diabetic patients and indicate THPO as a new mediator involved in the pathogenesis of this phenomenon, with potential therapeutic implications.

In order to study the contribution of THPO to increased platelet activation in type 1 diabetic patients, we evaluated the effects of adding plasma samples from diabetic patients to platelets of healthy subjects in vitro and inhibiting THPO biological activity by using rhTHPOR. In these experimental conditions, plasma from type 1 diabetic patients, but not from healthy subjects, enhanced platelet–monocyte and platelet–granulocyte binding in blood samples from healthy donors. The contribution of THPO to this priming effect is suggested by (1) the correlation analysis showing that THPO levels and leucocyte-platelet binding induced by patient plasma samples in whole blood consensually increased, and (2) the inhibitory effect of the rhTHPOR.

Taken together, our in vivo and in vitro results support the hypothesis that the elevated concentrations of THPO present in the circulation of diabetic patients may facilitate platelet activation by sensitizing platelets to the action of other agonists, thus augmenting the cardiovascular risk of these patients. This model is consistent with what we had previously observed in other clinical conditions characterized by increased thrombotic risk (acute coronary syndromes, sepsis, burn injury, cigarette smoking), as well as with the priming effect induced in vivo by THPO infusion in non-human primates [62].

Finally, we can speculate that this effect may be implicated in the pathogenesis of the “reduced sensitivity” of platelets from diabetic patients to oral antiplatelet agents used for primary and secondary prevention, a phenomenon that is particularly prevalent in diabetic patients [63,64,65] and could explain the reduced efficacy of aspirin treatment in this patient population [63,66,67,68].

In conclusion, our results suggest that increased THPO levels may enhance platelet activation in patients with type 1 diabetes mellitus and contribute to increased cardiovascular risk in this patient population.

## 4. Materials and Methods

### 4.1. Patients and Healthy Controls

This was a case–control study performed on 28 diabetic patients attending the Department of Endocrinology, Diabetology and Metabolism—Diabetes Center at A.O.U. Città della Salute e della Scienza di Torino, Italy. Twenty-eight healthy subjects matched in a 1:1 ratio for age and gender to cases were also enrolled. None of both diabetic patients and healthy subjects had acute or chronic infections, preproliferative or proliferative retinopathy, diabetic neuropathy, cardiovascular disease, hypertension, or drug treatment (except insulin in diabetic patients) at the time of enrolment.

Exclusion criteria were as follows: age < 18 years or >50 years; current smoker; treatment with regular medication (other than insulin for T1DM group) or anti-platelet agent assumption within the preceding 2 weeks; clinical evidence of any condition associated with increased levels of acute phase proteins (infectious diseases, surgery within the previous 1 month); known hematological diseases affecting coagulation, platelet, or THPO production; malignancies; body mass index > 30 kg/m^2^; hypercholesterolemia; hypertension; cardiovascular diseases; micro/macroalbuminuria; neuropathy; proliferative retinopathy; and renal or hepatic insufficiency.

The study was approved by the Institutional Ethics Committee of A.O.U. Città della Salute e della Scienza di Torino (#0076185). Written informed consent was obtained from all patients; all samples were anonymously coded in accordance with the Declaration of Helsinki.

### 4.2. Blood Collection Protocol

Venipuncture was performed with a 19-gauge butterfly infusion set, without venous stasis. After discarding the first 4 mL, blood was drawn into Vacutainers containing EDTA or 3.8% trisodium citrate, as appropriate.

To obtain plasma samples, EDTA-anticoagulated tubes were first centrifuged at 1600× *g* for 10 min at 4 °C, then at 12,500× *g* for 10 min at 4 °C, filtered through 0.22 µm pores, and immediately frozen and stored at −70 °C until analysis.

Platelet-rich plasma (PRP) was prepared by centrifugation of 3.8% trisodium citrate-anticoagulated blood for 15 min at 180× *g* [12].

### 4.3. Leucocyte–Platelet Adhesion and Platelet THPOR Expression Ex Vivo

Platelet–leukocyte aggregates were analyzed by three-color staining of whole blood samples, as previously described [15]. Briefly, blood was diluted 1:1 with Tyrodes’s HEPES-buffered saline (pH 7.4); added to a mixture of FITC-conjugated anti-CD45 (Beckman Coulter, Miami, FL, USA), ECD-conjugated anti-CD14 (Beckman Coulter), and PE-conjugated anti-CD41 (Dako Cytomation, Glostrup, Denmark) monoclonal antibodies; and incubated 15 min at room temperature. Cells were then fixed with 1% paraformaldehyde and resuspended in 0.5 mL of PBS, after removal of erythrocytes by hypotonic lysis. Samples were analyzed on the EPICS-XL flow cytometer (Coulter Corp, Hialeah, FL, USA) using adequate compensation for different fluorochromes. Total leukocytes were identified by their positive staining with anti-CD45, and lymphocyte, granulocyte, and monocyte populations were discriminated on the ground of CD45 versus side scatter. The percentage of leukocyte sub-groups co-expressing CD45-CD41 (granulocytes-platelets) or CD14-CD41 (monocytes-platelets) over the total population of leukocytes expressing CD45 or CD14 was used as an index of platelet–leukocyte adhesion [15]. P-selectin expression was evaluated in whole blood using a mixture of FITC-conjugated anti-CD62P/P-selectin (Ancell Corporation, Bayport, MN, USA) or the appropriate isotypic control, and PE-conjugated anti-CD41 monoclonal antibodies. Platelets were identified by their characteristic light scatter and the positive signal provided by the platelet marker PE-anti-CD41 monoclonal antibody.

Platelet THPOR surface expression was examined in fixed PRP using an anti-THPOR monoclonal antibody (R&D Systems Inc., Minneapolis, MN, USA), followed by Alexa Fluor 488-conjugated anti-mouse IgG monoclonal antibody (Molecular Probes, Eugene, OR, USA).

### 4.4. Leucocyte–Platelet Adhesion In Vitro

In order to explore the potential involvement of humoral mediators present in the plasma of diabetic patients in enhancing platelet activation, we tested in vitro the effect of plasma from diabetic patients and normal subjects on platelet–leukocyte adhesion in whole blood obtained from healthy donors.

For in vitro experiments, 100 µL of diluted blood from healthy adult donors was pre-incubated at 37 °C with 25 µL of plasma of healthy or T1DM subjects for 5 min and then stimulated with EPI (4 µmol/L; Helena Laboratories, Beaumont, TX, USA). Samples were then processed and analyzed as described above. In separate experiments, the plasma was incubated with rhTHPOR (2.5 µg/mL; R&D Systems Inc.) for 5 min at 37 °C; the mixture of sample and rhTHPOR was added to whole blood, further incubated for 5 min at 37 °C and stimulated with EPI.

### 4.5. Biochemical Analyses

Plasma glucose was assayed by the glucose oxidase method (Beckman II, Glucose Analyser, Fullerton CA); HbAlc was determined by high-performance liquid chromatography (DIAMAT, Bio-Rad, Richmond, CA, USA); plasma triglycerides and total cholesterol were determined enzymatically (Boehringer Mannheim, Germany). High-density lipoprotein (HDL)-cholesterol was measured by precipitation with heparin and MgCl_2_ on whole plasma, whereas low-density lipoprotein (LDL)-cholesterol was calculated by Friedewald’s formula.

THPO levels in plasma were quantified using a specific ELISA kit (R&D Systems) following the manufacturer’s instructions. The lower detection limit of the assay was 7.45 pg/mL.

### 4.6. Statistical Analysis

Statistically significant differences between datasets were evaluated by the Mann–Whitney rank sum test or unpaired *t*-test, and paired *t*-tests, as appropriate. The Spearman correlation test was used to investigate the relationships between variables.

*p* < 0.05 was considered statistically significant.

Statistical analysis was performed with the GraphPad Prism 7 package (GraphPad Software, La Jolla, CA, USA).

## Figures and Tables

**Table 1:** Subject characteristics.

Characteristics	Controls (*n* = 28)	T1DM (*n* = 28)
Age (years), median (IQR)	26.00 (23.00–36.00)	28.00 (22.25–36.75)
Gender (*n*), female/male	10/18	10/18
WBC (10^9^/L), median (IQR)	5.38 (4.73–5.63)	6.15 (4.64–7.27)
Monocytes (10^9^/L), mean ± SE	0.37 ± 0.02	0.40 ± 0.04
Granulocytes (10^9^/L), median (IQR)	2.94 (2.56–3.39)	3.17 (2.07–4.78)
Platelets (10^9^/L), mean ± SE	231.60 ± 7.75	235.80 ± 14.58
MPV (fL), mean ± SE	10.42 ± 0.15	10.74 ± 0.20
PDW (fL), mean ± SE	12.52 ± 0.32	12.98 ± 0.37
Total cholesterol (mg/dL), mean ± SE	186.00 ± 5.88	175.40 ± 5.18
HDL cholesterol (mg/dL), mean ± SE	56.75 ± 2.72	51.36 ± 2.30
LDL cholesterol (mg/dL), mean ± SE	112.60 ± 6.10	111.40 ± 4.40
Triglycerides (mg/dL), median (IQR)	67.50 (57.25–81.50)	58.00 (48.00–78.00)
BMI (Kg/m^2^), mean ± SE	21.90 ± 0.59	21.57 ± 0.39
Diabetes duration (years), mean ± SE	n.a.	13.31 ± 1.55
Fasting glucose (mg/dL), median (IQR)	90.00 (85.25–100.80)	164.00 (130.00–217.00) ***
HbA1c (mmol/mol), median (IQR)	36.00 (34.00–38.00)	59.00 (51.25–66.75) ***

BMI = body mass index; HDL = high-density lipoprotein; IQR = interquartile range; LDL = low-density lipoprotein; MPV = mean platelet volume; n.a. = not applicable; PDW = platelet distribution width; T1DM = type 1 diabetes mellitus; WBC = white blood cell. *** *p* < 0.001 vs. controls, obtained by Mann–Whitney test.

## Data Availability

The data presented in this study are available on reasonable request from the corresponding author. The data are not publicly available due to privacy concerns.
